# Supplementary material for: Hasty sensorimotor decisions rely on an overlap of broad and selective changes in motor activity
Source: PLoS Biol. 2022 Apr 7;20(4):e3001598. doi: 10.1371/journal.pbio.3001598 (PMC9017893; doi:10.1371/journal.pbio.3001598)
Supplement: S1 Fig — Resting-state MEPs. (A) Resting-state recordings in a representative TMSFinger participant. As described in the Materials and methods section, participants performed the 2 block types (i.e., hasty and cautious context blocks) on separate experimental sessions. The blue and yellow traces represent raw MEP recordings, as obtained at rest in the hasty and cautious context sessions, respectively. In each session, the double-coil stimulation over the left and right finger representations allowed us to elicit MEPs in the index, the thumb and the pinky muscles of both hands at once. (B) Same as A for a TMSLeg participant. MEP amplitudes were smaller in the leg than in the finger representations, potentially due to the higher distance between the coil and the leg area, located in the interhemispheric fissure. Still, in each session, the stimulation over the left leg representations allowed us to elicit MEPs of reliable amplitudes in the right TA, as well as in the right lateral and medial heads of the gastrocnemius muscle at once. (C, D) Group-averaged resting-state excitability and statistical analysis. NS annotations indicate that the [rm]ANOVAs performed on resting-state MEPs did not show any significant difference between the hasty and cautious sessions, neither in TMSFinger participants (Effect of SESSION: F1,20 = 0.48, p = 0.497, partial η2 = 0.023; SESSION*REPRESENTATION interaction: F2,40 = 1.08, p = 0.348, partial η2 = 0.051), nor in TMSLeg participants (Effect of SESSION: F1,21 = 0.19, p = 0.663, partial η2 = 0.009; SESSION*REPRESENTATION interaction: F2,42 = 2.07, p = 0.138, partial η2 = 0.089). Further, a BF analysis provided substantial evidence for a lack of effect of the factor SESSION on resting-state MEPs (BFs = 5.58 and 5.30, in TMSFinger and TMSLeg participants, respectively). The hash signs above the bars indicate that MEP amplitudes were significantly higher than 0 in all muscles (all t-values > 5.5, all p-values < 0.0001 after Bonferroni correction). E [file pbio.3001598.s001.docx]

**
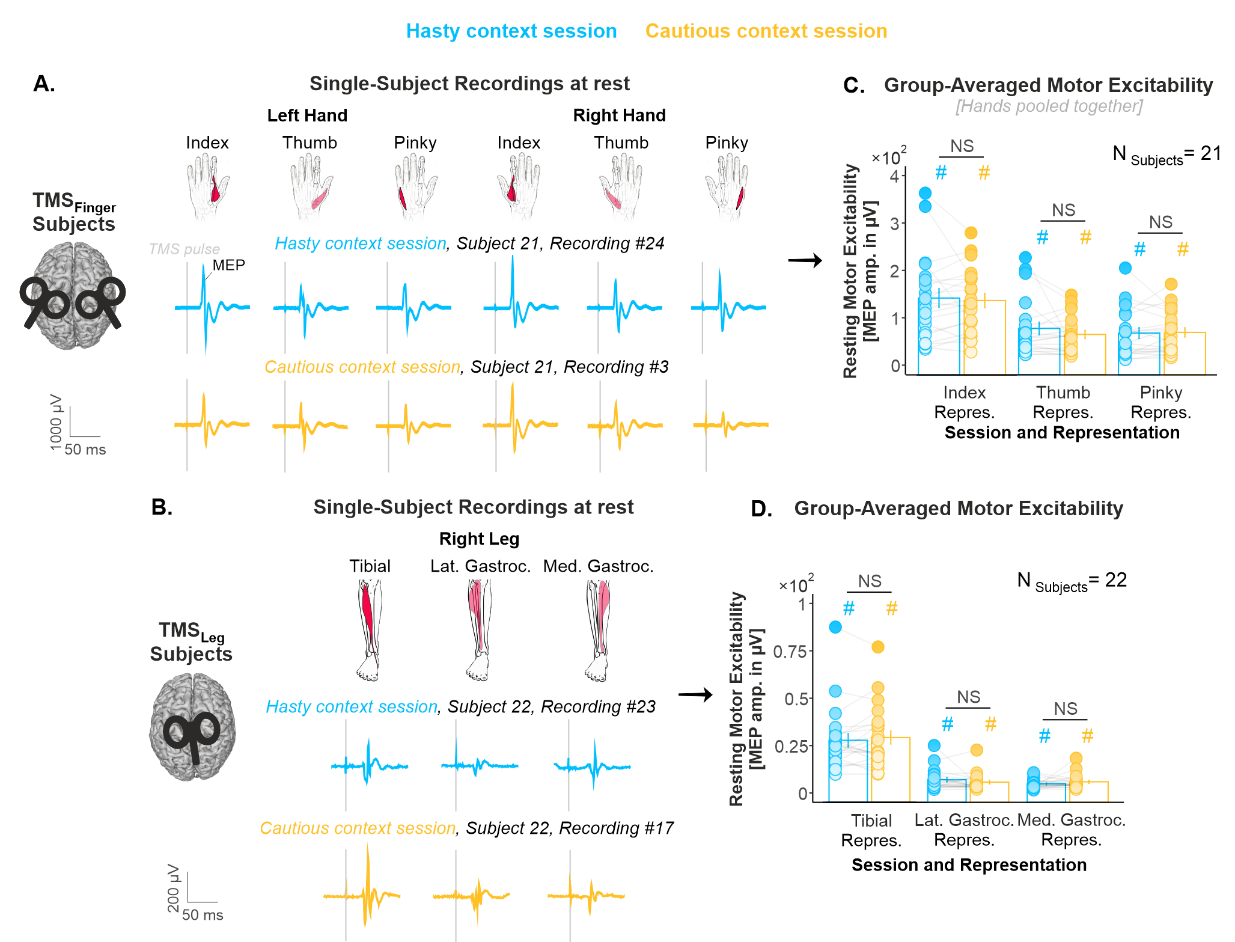
**

**S1 Fig (related to Fig 1.C): Resting-state MEPs. A. Resting-state recordings in a representative TMS_Finger_ subject.** As described in the Methods section, subjects performed the two block types (*i.e.*, hasty and cautious context blocks) on separate experimental sessions. The blue and yellow traces represent raw MEP recordings, as obtained at rest in the hasty and cautious context sessions, respectively. In each session, the double-coil stimulation over the left and right finger representations allowed us to elicit MEPs in the index, the thumb and the pinky muscles of both hands at once. **B. Same as A for a TMS_Leg_ subject.** MEP amplitudes were smaller in the leg than in the finger representations, potentially due to the higher distance between the coil and the leg area, located in the interhemispheric fissure. Still, in each session, the stimulation over the left leg representations allowed us to elicit MEPs of reliable amplitudes in the right tibialis anterior, as well as in the right lateral and medial heads of the gastrocnemius muscle at once. **C and D.** **Group-averaged resting-state excitability and statistical analysis.** NS annotations indicate that the repeated-measures [rm]ANOVAs performed on resting-state MEPs did not show any significant difference between the hasty and cautious sessions, neither in TMS_Finger_ subjects (Effect of SESSION: F_1,20_ = 0.48, p = .497, partial η^2^ = .023; SESSION*REPRESENTATION interaction: F_2,40_ = 1.08, p = .348, partial η^2^ = .051), nor in TMS_Leg_ subjects (Effect of SESSION: F_1,21_ = 0.19, p = .663, partial η^2^ = .009; SESSION*REPRESENTATION interaction: F_2,42_ = 2.07, p = .138, partial η^2^ = .089). Further, a Bayes Factor (BF) analysis provided substantial evidence for a lack of effect of the factor SESSION on resting-state MEPs (BFs = 5.58 and 5.30, in TMS_Finger_ and TMS_Leg_ subjects, respectively). The hash signs above the bars indicate that MEP amplitudes were significantly higher than 0 in all muscles (all t-values > 5.5, all p-values < .0001 after Bonferroni correction). Error bars represent 1 SEM. All individual and group-averaged numerical data exploited for S1 Fig are freely available at this link <https://osf.io/tbw7h/> (‘Fig_S1_Data.xlsx’).

Altogether, these data show that the two TMS protocols allowed us to record MEPs that were both reproducible across sessions and of reliable amplitudes in all of the investigated muscles.
