# Supplementary material for: Hasty sensorimotor decisions rely on an overlap of broad and selective changes in motor activity
Source: PLoS Biol. 2022 Apr 7;20(4):e3001598. doi: 10.1371/journal.pbio.3001598 (PMC9017893; doi:10.1371/journal.pbio.3001598)
Supplement: S2 Fig — The context-dependent shift in decision behavior was comparable in the 3 TMS subgroups (i.e., TMSFinger [top panel] and TMSLeg [middle panel] and No-TMS participants [bottom panel]). (A) DTs. We performed a rmANOVA while considering TMS-SUBGROUP as a categorical predictor. We did not find any significant effect of TMS-SUBGROUP (F2,47 = 1.39, p = 0.258, partial η2 = 0.056) nor of its interaction with the factor CONTEXT on DTs (F2,47 = 1.03, p = 0.362, partial η2 = 0.042). Further, a BF analysis provided substantial evidence for a lack of effect of the TMS-SUBGROUP on DTs (BF = 4.06). (B) Same as A. for decision accuracy. There was no significant effect of TMS-SUBGROUP (F2,47 = 1.05, p = 0.357, partial η2 = 0.043) nor of its interaction with the factor CONTEXT on accuracy (F2,47 = 0.38, p = 0.687, partial η2 = 0.016). The BF was of 4.07 for the effect of the TMS-SUBGROUP, revealing substantial evidence for a lack of effect of this factor on accuracy. (C) Urgency functions. There was also no significant effect of TMS-SUBGROUP (F2,47 = 0.89, p = 0.415, partial η2 = 0.037) nor of its interaction with the factor CONTEXT on the slope of the urgency functions (F2,47 = 0.81, p = 0.452, partial η2 = 0.033). Similarly, there was no significant effect of TMS-SUBGROUP (F2,47 = 0.19, p = 0.820, partial η2 = 0.008) nor of its interaction with the factor CONTEXT on the intercept of the functions (F2,47 = 0.44, p = 0.643, partial η2 = 0.018). Here again, BFs showed substantial evidence for a lack of effect of the TMS-SUBGROUP on the slope and the intercept of the functions (BFs = 4.22 and 8.35, respectively). Error bars represent 1 SEM. All individual and group-averaged numerical data exploited for S2 Fig are freely available at this link: https://osf.io/tbw7h. Overall, these results highlight that the 3 subgroups presented very similar effects of context on all of these behavioral variables, indicating that the application of TMS over the finger and leg representations did not pert [file pbio.3001598.s002.docx]

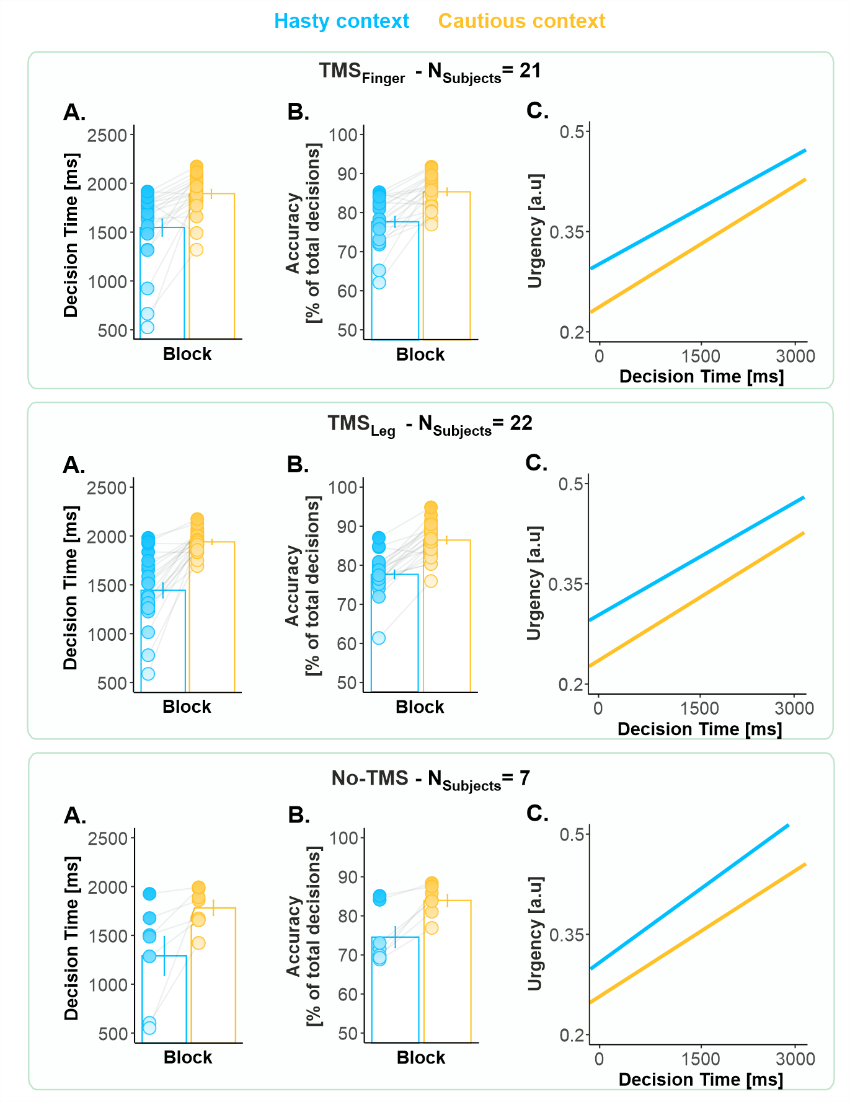


**S2 Fig (related to Fig 2)**: **The context-dependent shift in decision behavior was comparable in the three TMS subgroups (*i.e.*, TMS_Finger_ [top panel], TMS_Leg_ [middle panel] and No-TMS subjects [bottom panel]).** **A. Decision times.** We performed a rmANOVA while considering TMS-SUBGROUP as a categorical predictor. We did not find any significant effect of TMS-SUBGROUP (F_2,47_ = 1.39, p = .258, partial η^2^ = .056) nor of its interaction with the factor CONTEXT on DTs (F_2,47_ = 1.03, p = .362, partial η^2^ = .042). Further, a BF analysis provided substantial evidence for a lack of effect of the TMS-SUBGROUP on DTs (BF = 4.06). **B. Same as A. for decision accuracy.** There was no significant effect of TMS-SUBGROUP (F_2,47_ = 1.05, p = .357, partial η^2^ = .043) nor of its interaction with the factor CONTEXT on accuracy (F_2,47_ = 0.38, p = .687, partial η^2^ = .016). The BF was of 4.07 for the effect of the TMS-SUBGROUP, revealing substantial evidence for a lack of effect of this factor on accuracy. **C. Urgency functions.** There was also no significant effect of TMS-SUBGROUP (F_2,47_ = 0.89, p = .415, partial η^2^ = .037) nor of its interaction with the factor CONTEXT on the slope of the urgency functions (F_2,47_ = 0.81, p = .452, partial η^2^ = .033). Similarly, there was no significant effect of TMS-SUBGROUP (F_2,47_ = 0.19, p = .820, partial η^2^ = .008) nor of its interaction with the factor CONTEXT on the intercept of the functions (F_2,47_ = 0.44, p = .643, partial η^2^ = .018). Here again, BFs showed substantial evidence for a lack of effect of the TMS-SUBGROUP on the slope and the intercept of the functions (BFs = 4.22 and 8.35, respectively). Error bars represent 1 SEM. All individual and group-averaged numerical data exploited for S2 Fig are freely available at this link <https://osf.io/tbw7h/> (‘Fig_S2_Data.xlsx’).

Overall, these results highlight that the three subgroups presented very similar effects of context on all of these behavioral variables, indicating that the application of TMS over the finger and leg representations did not perturb SAT regulation in our task.
