# Supplementary material for: Hasty sensorimotor decisions rely on an overlap of broad and selective changes in motor activity
Source: PLoS Biol. 2022 Apr 7;20(4):e3001598. doi: 10.1371/journal.pbio.3001598 (PMC9017893; doi:10.1371/journal.pbio.3001598)
Supplement: S3 Fig — Trial selection and RT-matching procedure for MEP analysis. Given the between-context difference in decision speed (see Fig 2 and S1 Fig) and its potential effect on MEP amplitudes, we adopted a RT-matching procedure to homogenize RT distributions across contexts (see [1] for a similar procedure). The procedure consisted in discretizing each participant’s RT distributions into bins of 200 ms width and, for each bin, randomly selecting a matched number of trials. To do so, for each bin, we kept all the trials of the context condition that had the lowest trial count and selected a matched number from the context condition that had the greatest trial count in that bin. As a result, the MEPs included in the analysis were those for which the RT distributions for the hasty and cautious overlapped (gray area on single-participant distributions). In a few participants (for whom the distribution overlap was very small), this procedure led to the exclusion of many trials. Hence, we had to exclude 2 and 6 participants out of the 21 TMSFinger and 22 TMSLeg participants as they presented too few trials after this procedure for each timing and context (i.e., <8 trials on average). On the remaining 19 TMSFinger and 16 TMSLeg participants, the included trials involved comparable RTs in the hasty and cautious contexts, both in TMSFinger participants (2,230 ± 39 ms and 2,230 ± 38 ms, respectively) and in TMSLeg participants (2,266 ± 27 ms and 2,278 ± 26 ms, respectively; see bar graphs). Error bars represent 1 SEM. All individual and group-averaged numerical data exploited for S3 Fig are freely available at this link: https://osf.io/tbw7h. Hence, this procedure guaranteed that any effect of context on MEP amplitudes could not result from a between-context difference in RT in the included trials. MEP, motor-evoked potential; RT, reaction time; TMS, transcranial magnetic stimulation. (DOCX) [file pbio.3001598.s003.docx]

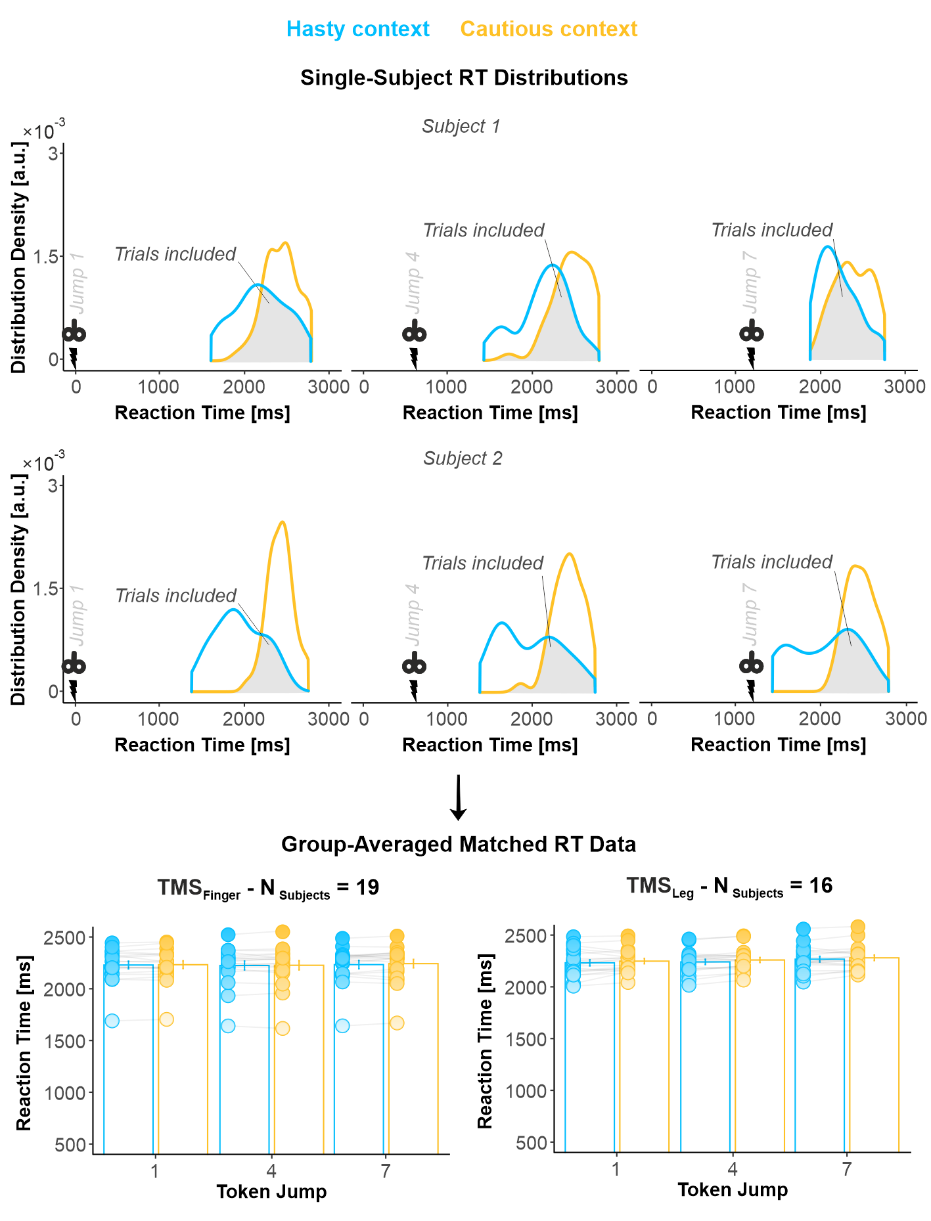


**S3 Fig (related to Fig 3)**: **Trial selection and RT-matching procedure for MEP analysis.** Given the between-context difference in decision speed (see Fig 2 and S2) and its potential effect on MEP amplitudes, we adopted a RT-matching procedure to homogenize RT distributions across contexts (see Murphy et al., 2016, Nat. Comm., for a similar procedure). The procedure consisted in discretizing each subject’s RT distributions into bins of 200 ms width and, for each bin, randomly selecting a matched number of trials. To do so, for each bin, we kept all the trials of the context condition that had the lowest trial count and selected a matched number from the context condition that had the greatest trial count in that bin. As a result, the MEPs included in the analysis were those for which the RT distributions for the hasty and cautious overlapped (grey area on single-subject distributions). In a few subjects (for whom the distribution overlap was very small), this procedure led to the exclusion of many trials. Hence, we had to exclude 2 and 6 subjects out of the 21 TMS_Finger_ and 22 TMS_Leg_ participants as they presented too few trials after this procedure for each timing and context (*i.e.*, < 8 trials on average). On the remaining 19 TMS_Finger_ and 16 TMS_Leg_ subjects, the included trials involved comparable RTs in the hasty and cautious contexts, both in TMS_Finger_ subjects (2230 ± 39 ms and 2230 ± 38 ms, respectively) and in TMS_Leg_ subjects (2266 ± 27 ms and 2278 ± 26 ms, respectively; see bar graphs). Error bars represent 1 SEM. All individual and group-averaged numerical data exploited for S3 Fig are freely available at this link <https://osf.io/tbw7h/> (‘Fig_S3_Data.xlsx’).

Hence, this procedure guaranteed that any effect of context on MEP amplitudes could not result from a between-context difference in RT in the included trials.
