# Supplementary material for: Hasty sensorimotor decisions rely on an overlap of broad and selective changes in motor activity
Source: PLoS Biol. 2022 Apr 7;20(4):e3001598. doi: 10.1371/journal.pbio.3001598 (PMC9017893; doi:10.1371/journal.pbio.3001598)
Supplement: S4 Fig — The context-dependent shift in decision behavior was present in the participants included in the MEP analysis. As mentioned in the Results and in the Materials and methods sections, we had to exclude 3 out of the 21 TMSFinger participants and 6 out of the 22 TMSLeg participants following the RT-matching procedure. Participants excluded following this procedure were more likely to present a too small overlap between their RT distributions and thus to exhibit strong SAT shifts. To ensure that the participants included in the MEP analysis presented a SAT shift, we performed a statistical analysis on their behavioral data. A between-context comparison revealed that DTs and accuracy were significantly lower in the hasty context (TMSFinger and TMSLeg participants pooled together: t34 = −7.69, p < 0.0001, Cohen’s d = 1.304 and t34 = −9.25, p < 0.0001, Cohen’s d = 1.565, respectively; panel A and B). Besides, the urgency intercept was significantly higher in the hasty relative to the cautious context (t49 = 5.37, p < 0.0001, Cohen’s d = 0.909; panel C). All individual and group-averaged numerical data exploited for S4 Fig are freely available at this link: https://osf.io/tbw7h. DT, decision time; MEP, motor-evoked potential; RT, reaction time; SAT, speed–accuracy trade-off; TMS, transcranial magnetic stimulation. (DOCX) [file pbio.3001598.s004.docx]

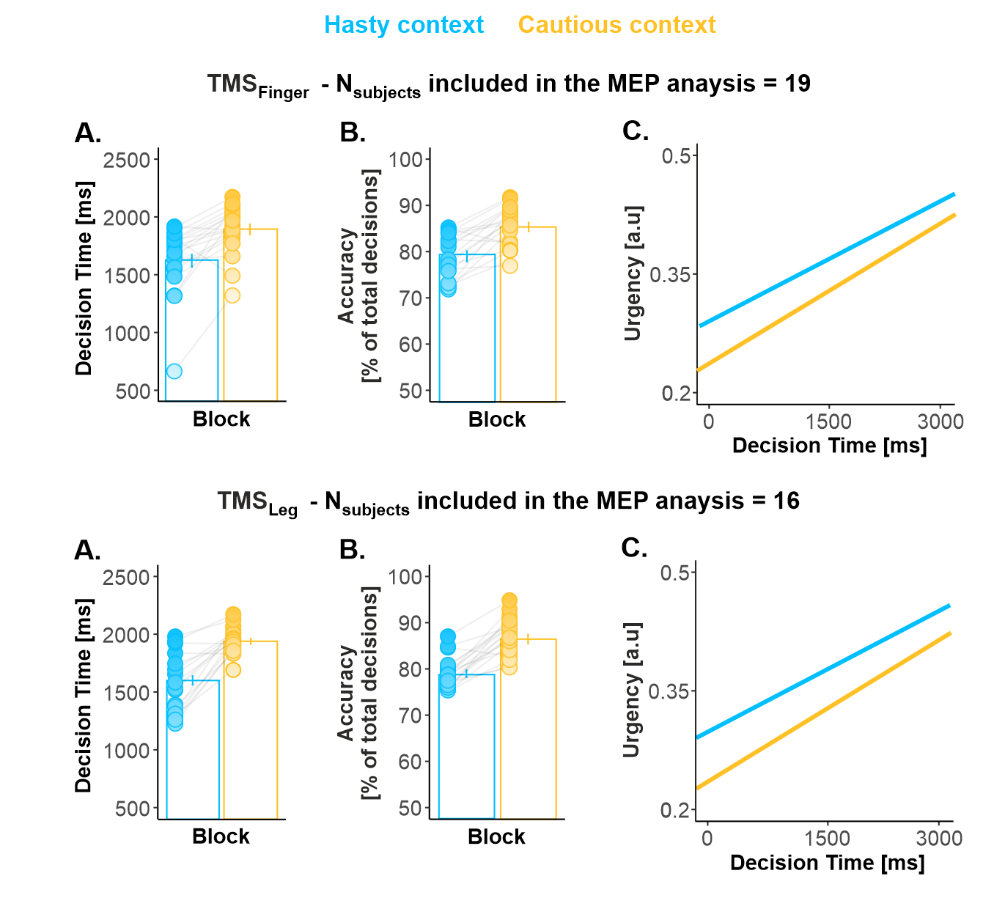


**S4 Fig (related to Fig 4)**: **The context-dependent shift in decision behavior was present in the subjects included in the MEP analysis.** As mentioned in the Results and Methods sections, we had to exclude 3 out of the 21 TMS_Finger_ subjects and 6 out of the 22 TMS_Leg_ subjects following the RT-matching procedure. Subjects excluded following this procedure were more likely to present a too small overlap between their RT distributions and thus to exhibit strong SAT shifts. To ensure that the subjects included in the MEP analysis presented a SAT shift, we performed a statistical analysis on their behavioral data. A between-context comparison revealed that DTs and accuracy were significantly lower in the hasty context (TMS_Finger_ and TMS_Leg_ subjects pooled together: t_34_ = -7.69, p < .0001, Cohen’s d = 1.304 and t_34_ = -9.25, p < .0001, Cohen’s d = 1.565, respectively; panel A and B). Besides, the urgency intercept was significantly higher in the hasty relative to the cautious context (t_49_ = 5.37, p < .0001, Cohen’s d = 0.909; panel C). All individual and group-averaged numerical data exploited for S4 Fig are freely available at this link <https://osf.io/tbw7h/> (‘Fig_S4_Data.xlsx’).
