# Supplementary material for: Hasty sensorimotor decisions rely on an overlap of broad and selective changes in motor activity
Source: PLoS Biol. 2022 Apr 7;20(4):e3001598. doi: 10.1371/journal.pbio.3001598 (PMC9017893; doi:10.1371/journal.pbio.3001598)
Supplement: S5 Fig — The broad amplification affected the 3 leg representations of the chosen side in a reproducible way. (A) Effect of CONTEXT on motor excitability on the chosen side. Excitability changes were more variable in the leg than in the finger representations (i.e., compared to Fig 4), potentially due to the smaller MEP amplitudes obtained for the leg representation (see S1 Fig). Despite this variability, the effect of context was comparable in the 3 investigated leg representations, with higher excitability values in the hasty than in the cautious context. As such, there was no significant CONTEXT*REPRESENTATION interaction (F2,30 = 0.53, p = 0.595, partial η2 = 0.034), nor any CONTEXT*TIMING*REPRESENTATION interaction (F4,60 = 1.53, p = 0.202, partial η2 = 0.093); BF for the latter analysis was of 14.03, providing strong evidence for a lack of effect on this interaction. *: significant effect of context at p < 0.05. (B) Same as A. for the unchosen side. Here again, the 3 leg representations exhibited similar patterns of excitability changes, with no evident impact of context and an overall rise as time elapsed. Indeed, there was no significant CONTEXT*REPRESENTATION interaction (F2,30 = 0.58, p = 0.561, partial η2 = 0.037), nor any CONTEXT*TIMING*REPRESENTATION interaction (F4,60 = 0.47, p = 0.756, partial η2 = 0.030); BF for the latter analysis was of 17.65, providing strong evidence for a lack of effect on this interaction. Error bars represent 1 SEM. All individual and group-averaged numerical data exploited for S5 Fig are freely available at this link: https://osf.io/tbw7h. BF, Bayes factor; MEP, motor-evoked potential. (DOCX) [file pbio.3001598.s005.docx]

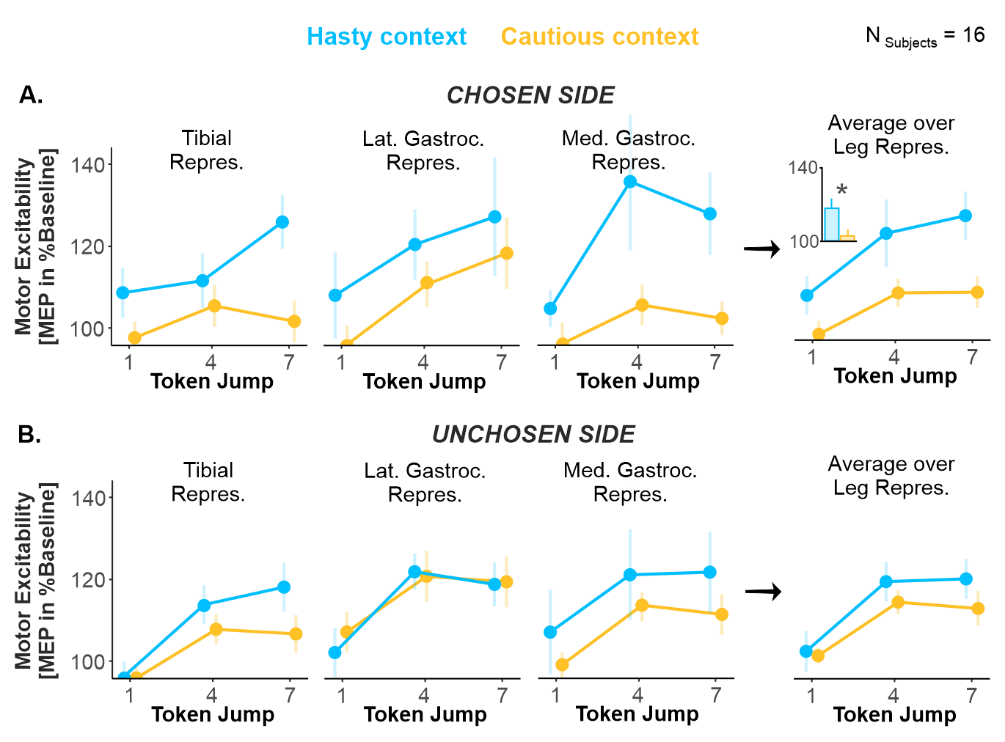


**S2 Fig (related to Fig 4)**: **The broad amplification affected the three leg representations of the chosen side in a reproducible way. A. Effect of CONTEXT on motor excitability on the chosen side.** Excitability changes were more variable in the leg than in the finger representations (*i.e.*, compared to Fig 4), potentially due to the smaller MEP amplitudes obtained for the leg representation (see Fig S1). Despite this variability, the effect of context was comparable in the three investigated leg representations, with higher excitability values in the hasty than in the cautious context. As such, there was no significant CONTEXT*REPRESENTATION interaction (F_2,30_ = 0.53, p = .595, partial η^2^ = .034), nor any CONTEXT*TIMING*REPRESENTATION interaction (F_4,60_ = 1.53, p = .202, partial η^2^ = .093); BF for the latter analysis was of 14.03, providing strong evidence for a lack of effect on this interaction. *: significant effect of context at p < .05. **B. Same as A. for the unchosen side.** Here again, the three leg representations exhibited similar patterns of excitability changes, with no evident impact of context and an overall rise as time elapsed. Indeed, there was no significant CONTEXT*REPRESENTATION interaction (F_2,30_ = 0.58, p = .561, partial η^2^ = .037), nor any CONTEXT*TIMING*REPRESENTATION interaction (F_4,60_ = 0.47, p = .756, partial η^2^ = .030); BF for the latter analysis was of 17.65, providing strong evidence for a lack of effect on this interaction. Error bars represent 1 SEM. All individual and group-averaged numerical data exploited for S2 Fig are freely available at this link <https://osf.io/tbw7h/> (‘Fig_S2_Data.xlsx’).
