# Supplementary material for: Hasty sensorimotor decisions rely on an overlap of broad and selective changes in motor activity
Source: PLoS Biol. 2022 Apr 7;20(4):e3001598. doi: 10.1371/journal.pbio.3001598 (PMC9017893; doi:10.1371/journal.pbio.3001598)
Supplement: S6 Fig — The effects of context were still present when exploiting the full, RT-unmatched dataset. The RT-matching procedure described in S3 Fig ensured similar RTs between the 2 contexts, but it raises a potential confound by emphasizing the slowest trials from the hasty context and the fastest trials from the cautious context. However, concerns about that confound are reduced by the observation that the same analyses performed on the full set of trials, without RT matching, produced the same results. All individual and group-averaged numerical data exploited for S6 Fig are freely available at this link: https://osf.io/tbw7h. RT, reaction time. (DOCX) [file pbio.3001598.s006.docx]

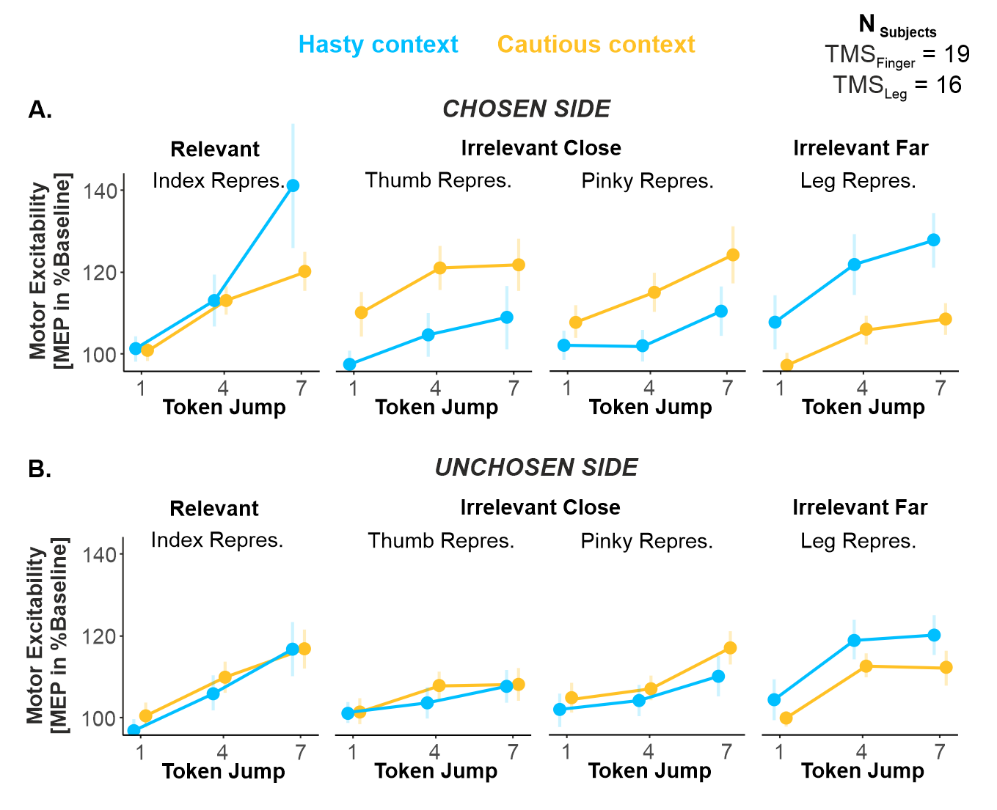


**S6 Fig (related to Fig 4): The effects of context were still present when exploiting the full, RT-unmatched dataset.** The RT-matching procedure described in S3 Fig ensured similar reaction times between the two contexts, but it raises a potential confound by emphasizing the slowest trials from the hasty context and the fastest trials from the cautious context. However, concerns about that confound are reduced by the observation that the same analyses performed on the full set of trials, without RT-matching, produced the same results. All individual and group-averaged numerical data exploited for S6 Fig are freely available at this link <https://osf.io/tbw7h/> (‘Fig_S6_Data.xlsx’).
