# Supplementary material for: Hasty sensorimotor decisions rely on an overlap of broad and selective changes in motor activity
Source: PLoS Biol. 2022 Apr 7;20(4):e3001598. doi: 10.1371/journal.pbio.3001598 (PMC9017893; doi:10.1371/journal.pbio.3001598)
Supplement: S7 Fig — Single-trial correlations. Example of 4 correlations obtained from the single-trial analysis. We pooled the trials of all participants together (normalized to baseline; NParticipants = 16), providing us with a large pool of data points (NTrials = 528) and applied a rmCorr analysis. RmCorr accounts for nonindependence among observations using ANCOVA to statistically adjust for interindividual variability. By removing measured variance between participants, rmCorr provides the best linear fit for each participant using parallel regression lines (the same slope) with varying intercepts, as can be seen in each cloud of points. As indicated in the Materials and methods section, normalized single-trial data were squared root-transformed to enhance the normality of the distributions (although similar findings were obtained on nontransformed data). As evident on this figure, excitability changes in the chosen index representation positively covaried with changes in other finger representations (here, the thumb and pinky representations of the chosen side). Further, while the strength of the correlation between the index and the thumb representation was comparable in the hasty and cautious contexts (R = 0.63 and 0.56, 95% CIs = [0.58 0.68] and [0.49 0.61], respectively), the correlation between the index and the pinky representation was significantly weaker in the former than the latter context (R = 0.08 and R = 0.34, 95% CIs = [−0.007 016] and [0.27 0.42], respectively). A similar decorrelation was observed between the chosen index and the index and thumb representations of the unchosen side (see Fig 6 in the main text). All individual and group-averaged numerical data exploited for S7 Fig are freely available at this link: https://osf.io/tbw7h. ANCOVA, analysis of covariance; rmCorr, repeated measures correlation. (DOCX) [file pbio.3001598.s007.docx]

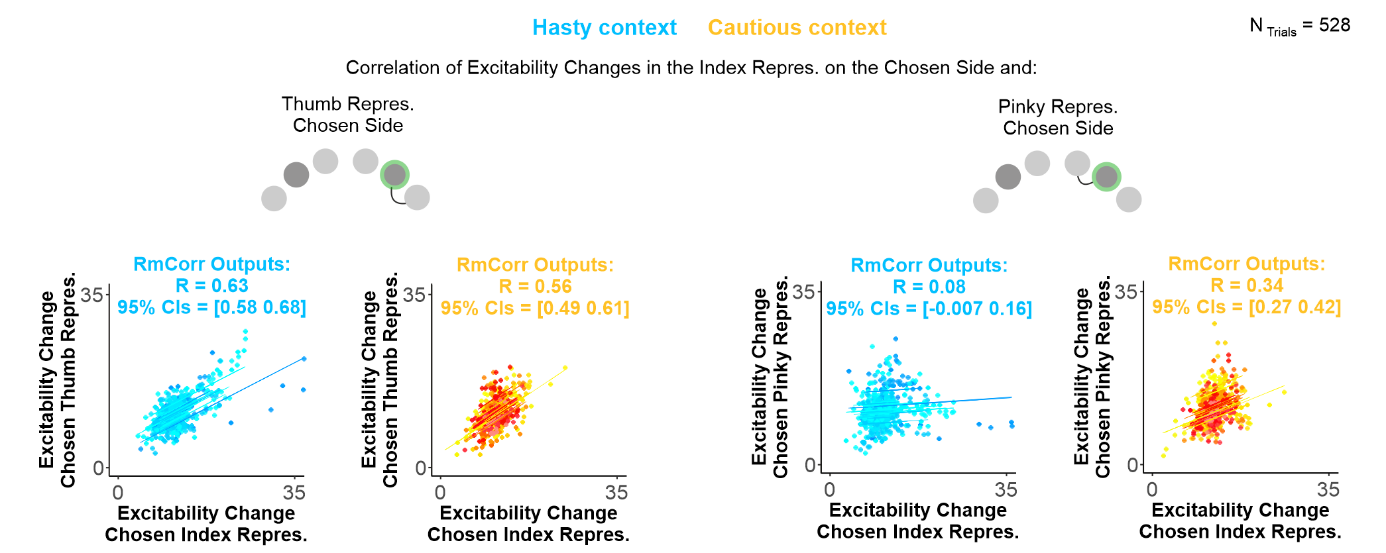


**S7 Fig (related to Fig 6): Single-trial correlations.** Example of four correlations obtained from the single-trial analysis. We pooled the trials of all subjects together (normalized to baseline; N_Subjects_ = 16), providing us with a large pool of data points (N_Trials_ = 528) and applied a repeated-measures correlation (rmCorr) analysis. RmCorr accounts for non-independence among observations using analysis of covariance (ANCOVA) to statistically adjust for inter-individual variability. By removing measured variance between-participants, rmCorr provides the best linear fit for each participant using parallel regression lines (the same slope) with varying intercepts, as can be seen in each cloud of points. As indicated in the methods section, normalized single-trial data were squared root-transformed to enhance the normality of the distributions (although similar findings were obtained on non-transformed data). As evident on this Fig, excitability changes in the chosen index representation positively co-varied with changes in other finger representations (here the thumb and pinky representations of the chosen side). Further, while the strength of the correlation between the index and the thumb representation was comparable in the hasty and cautious contexts (R = 0.63 and 0.56, 95 % CIs = [0.58 0.68] and [0.49 0.61], respectively), the correlation between the index and the pinky representation was significantly weaker in the former than the latter context (R = 0.08 and R = 0.34, 95 % CIs = [-0.007 016] and [0.27 0.42], respectively). A similar decorrelation was observed between the chosen index and the index and thumb representations of the unchosen side (see Fig 6 in the main text). All individual and group-averaged numerical data exploited for S7 Fig are freely available at this link <https://osf.io/tbw7h/> (‘Fig_6&S7_Data.xlsx’).
