# Supplementary material for: Hasty sensorimotor decisions rely on an overlap of broad and selective changes in motor activity
Source: PLoS Biol. 2022 Apr 7;20(4):e3001598. doi: 10.1371/journal.pbio.3001598 (PMC9017893; doi:10.1371/journal.pbio.3001598)
Supplement: S1 Table — The context-dependent shift in SAT did not depend on the session. As mentioned in the Results section, the session order was not completely counterbalanced among the 50 participants included in the behavioral analysis: 24 participants started the experiment with the hasty session while 26 started with the cautious one. To ensure that the effects of CONTEXT observed on DT, accuracy, urgency intercept reported in Fig 2 did not depend on the lack of counterbalancing, we performed Bayesian rmANOVAs, testing whether any of these effects interacted with the factor SESSION ORDER. We did not find any significant CONTEXT*SESSION ORDER interaction, whether looking at DTs (F1, 48 = 0.00043, p = 0.984, partial η2 = 8.81×10^−5^), at accuracy (F1, 48 = 1.30, p = 0.259, partial η2 = 0.026), or at urgency intercepts (F1, 48 = 1.30, p = 0.259, partial η2 = 0.055). BFs for these 3 variables were 3.48, 4.45 and 3.22, providing strong evidence for a lack of CONTEXT*SESSION ORDER interaction on these variables. BF, Bayes factor; DT, decision time; rmANOVA, repeated measures analyses of variance; SAT, speed–accuracy trade-off. (DOCX) [file pbio.3001598.s009.docx]

| **Interaction tested** | **Key statistics** | **DT** | **Accuracy** | | **Urgency Intercept** |
| --- | --- | --- | --- | --- | --- |
| **CONTEXT * SESSION-ORDER** | F-value | 0.0004 | 1.30 | 2.82 | |
|  | p-value | .984 | .259 | .099 | |
|  | **Bayes Factor** | **3.48** | **4.45** | **3.22** | |

**S1 Table (related to Fig 2): The context-dependent shift in SAT did not depend on the session.** As mentioned in the Results section, the session order was not completely counterbalanced among the 50 subjects included in the behavioral analysis: 24 subjects started the experiment with the hasty session while 26 started with the cautious one. To ensure that the effects of CONTEXT observed on DT, accuracy, urgency intercept reported in Fig 2 did not depend on the lack of counterbalancing, we performed Bayesian rmANOVAs, testing whether any of these effects interacted with the factor SESSION-ORDER. We did not find any significant CONTEXT*SESSION-ORDER interaction, whether looking at DTs (F_1, 48_ = 0.00043, p = .984, partial η^2^ = 8.81×10^-5^), at accuracy (F_1, 48_ = 1.30, p = .259, partial η^2^ = .026), or at urgency intercepts (F_1, 48_ = 1.30, p = .259, partial η^2^ = .055). Bayes Factors for these three variables were 3.48, 4.45 and 3.22, providing strong evidence for a lack of CONTEXT*SESSION-ORDER interaction on these variables.
