# Supplementary material for: Hasty sensorimotor decisions rely on an overlap of broad and selective changes in motor activity
Source: PLoS Biol. 2022 Apr 7;20(4):e3001598. doi: 10.1371/journal.pbio.3001598 (PMC9017893; doi:10.1371/journal.pbio.3001598)
Supplement: S2 Table — Decomposition of the CONTEXT*TIMING*REPRESENTATION interaction using separate rmANOVAs for each representation with CONTEXT and TIMING as within-participant factors. For the sake of homogeneity, the same procedure was applied throughout the manuscript to decompose significant effects following interactions. That is, Tukey HSD post hoc tests were applied for all pairs of conditions comprised in the interaction, and the correction was thus proportional to the number of pairs tested. Here, we exploited an alternative decomposition of the CONTEXT*TIMING*REPRESENTATION interaction found on the chosen side of TMSFinger participants, using 3 separate rmANOVAs for each representation with CONTEXT and TIMING as within-participant factors. First, this analysis showed that the global effect of TIMING reported in the manuscript (Fig 3) could be replicated for the 3 representations using this approach. Further, a CONTEXT*TIMING interaction was present in the index representation (F2,36 = 3.94, p = 0.028, partial η2 = 0.181), consistent with the effects reported in the manuscript (Fig 4A, left panel). However, this interaction was not present for the surrounding finger representations, neither for the thumb (F2,36 = 0.44, p = 0.646, partial η2 = 0.024) nor for the pinky one (F2,36 = 0.624, p = 0.543, partial η2 = 0.033). Interestingly, the thumb representation presented a main effect of CONTEXT (marginally significant: F1,18 = 4.34, p = 0.051, partial η2 = 0.194), while this effect was not significant for the pinky representation (F1,18 = 1.82, p = 0.194, partial η2 = 0.089). Altogether, this analysis may suggest that the surround suppression effect reported in the manuscript was the strongest in the thumb representation, putatively due to its stronger functional link with the index representation. The analysis also hints that, in the thumb representation, the suppression of excitability did not necessarily depend on the timing at which it was probed and was potentially already p [file pbio.3001598.s010.docx]

| **Effect tested** | **Key statistics** | **Motor excitability on the chosen side** | | |
| --- | --- | --- | --- | --- |
|  |  | **Index** | **Thumb** | **Pinky** |
| **CONTEXT** | F-value | 1.17 | **4.34** | 1.82 |
|  | p-value | .294 | **.051** | .194 |
|  |  |  |  |  |
| **TIMING** | F-value | **13.16** | **3.38** | **6.91** |
|  | p-value | **.00005** | **.045** | **.003** |
|  |  |  |  |  |
| **CONTEXT * TIMING** | F-value | **3.94** | 0.44 | 0.62 |
|  | p-value | **.028** | .646 | .543 |
|  |  |  |  |  |

**S2 Table (related to Fig 4): Decomposition of the CONTEXT*TIMING*REPRESENTATION interaction using separate rmANOVAs for each representation with CONTEXT and TIMING as within-subject factors.** For the sake of homogeneity, the same procedure was applied throughout the manuscript to decompose significant effects following interactions. That is, Tukey HSD post-hoc tests were applied for all pairs of conditions comprised in the interaction, and the correction was thus proportional to the number of pairs tested. Here, we exploited an alternative decomposition of the CONTEXT*TIMING*REPRESENTATION interaction found on the chosen side of TMS_Finger_ subjects, using three separate rmANOVAs for each representation with CONTEXT and TIMING as within-subject factors. First, this analysis showed that the global effect of TIMING reported in the manuscript (Fig 3) could be replicated for the three representations using this approach. Further, a CONTEXT*TIMING interaction was present in the index representation (F_2,36_ = 3.94, p = .028, partial η^2^ = .181), consistent with the effects reported in the manuscript (Fig 4.A, left panel). However, this interaction was not present for the surrounding finger representations, neither for the thumb (F_2,36_ = 0.44, p = .646, partial η^2^ = .024) nor for the pinky one (F_2,36_ = 0.624, p = .543, partial η^2^ = .033). Interestingly, the thumb representation presented a main effect of CONTEXT (marginally significant: F_1,18_ = 4.34, p = .051, partial η^2^ = .194), while this effect was not significant for the pinky representation (F_1,18_ = 1.82, p = .194, partial η^2^ = .089). Altogether, this analysis may suggest that the surround suppression effect reported in the manuscript was the strongest in the thumb representation, putatively due to its stronger functional link with the index representation. The analysis also hints that, in the thumb representation, the suppression of excitability did not necessarily depend on the timing at which it was probed and was potentially already present early on during the decision process (*i.e.*, see Jump_1_ in Fig 4.A).
