# Supplementary material for: Hasty sensorimotor decisions rely on an overlap of broad and selective changes in motor activity
Source: PLoS Biol. 2022 Apr 7;20(4):e3001598. doi: 10.1371/journal.pbio.3001598 (PMC9017893; doi:10.1371/journal.pbio.3001598)
Supplement: S3 Table — In TMSFinger participants, the effect of context on motor excitability observed on the chosen side did not depend on the session order. As mentioned in the Results section, the session order was not completely counterbalanced among the 19 TMSFinger participants included in the MEP analysis: 8 participants started the experiment with the hasty session, while 11 started with the cautious one. To ensure that the effects of CONTEXT observed on motor excitability in these participants did not depend on the lack of counterbalancing, we performed Bayesian rmANOVAs, testing whether the factor CONTEXT interacted with SESSION ORDER. We did not find any significant CONTEXT*SESSION ORDER (F1, 17 = 2.16, p = 0.159, partial η2 = 0.113), CONTEXT*REPRESENTATION *SESSION ORDER (F2, 34 = 1.48, p = 0.240, partial η2 = 0.081), CONTEXT*TIMING *SESSION ORDER (F2, 34 = 2.49, p = 0.097, partial η2 = 0.128), or CONTEXT*REPRESENTATION*TIMING*SESSION ORDER interaction (F4, 68 = 0.32, p = 0.863, partial η2 = 0.018). BFs for these interactions ranged between 3.08 and 30.09, providing strong to decisive evidence for a lack of effect of the session order on the effect of context on motor excitability. BF, Bayes factor; MEP, motor-evoked potential; rmANOVA, repeated measures analyses of variance; TMS, transcranial magnetic stimulation. (DOCX) [file pbio.3001598.s011.docx]

| **Interaction tested** | **Key statistics** | **Motor Excitability on the chosen side** |
| --- | --- | --- |
| **CONTEXT * SESSION-ORDER** | F-value | 2.16 |
|  | p-value | .159 |
|  | **Bayes Factor** | **3.08** |
|  |  |  |
| **CONTEXT * REPRESENTATION * SESSION-ORDER** | F-value | 1.48 |
|  | p-value | .240 |
|  | **Bayes Factor** | **30.09** |
|  |  |  |
| **CONTEXT * TIMING * SESSION-ORDER** | F-value | 2.49 |
|  | p-value | .097 |
|  | **Bayes Factor** | **8.63** |
|  |  |  |
| **CONTEXT * REPRESENTATION * TIMING * SESSION-ORDER** | F-value | 0.32 |
|  | p-value | .863 |
|  | **Bayes Factor** | **10.09** |

**S3 Table (related to Fig 4): In TMS_Finger_ subjects, the effect of context on motor excitability observed on the chosen side did not depend on the session order.** As mentioned in the Results section, the session order was not completely counterbalanced among the 19 TMS_Finger_ subjects included in the MEP analysis: 8 subjects started the experiment with the hasty session while 11 started with the cautious one. To ensure that the effects of CONTEXT observed on motor excitability in these subjects did not depend on the lack of counterbalancing, we performed Bayesian rmANOVAs, testing whether the factor CONTEXT interacted with SESSION-ORDER. We did not find any significant CONTEXT*SESSION-ORDER (F_1, 17_ = 2.16, p = .159, partial η^2^ = .113), CONTEXT*REPRESENTATION *SESSION-ORDER (F_2, 34_ = 1.48, p = .240, partial η^2^ = .081), CONTEXT*TIMING *SESSION-ORDER (F_2, 34_ = 2.49, p = .097, partial η^2^ = .128), or CONTEXT*REPRESENTATION*TIMING*SESSION-ORDER interaction (F_4, 68_ = 0.32, p = .863, partial η^2^ = .018). Bayes Factors for these interactions ranged between 3.08 and 30.09, providing strong to decisive evidence for a lack of effect of the session order on the effect of context on motor excitability.
