# Supplementary material for: Hasty sensorimotor decisions rely on an overlap of broad and selective changes in motor activity
Source: PLoS Biol. 2022 Apr 7;20(4):e3001598. doi: 10.1371/journal.pbio.3001598 (PMC9017893; doi:10.1371/journal.pbio.3001598)
Supplement: Attachment — Submitted filename: Derosiere_Thura_Cisek_Duque_Rebuttal.docx [file pbio.3001598.s012.docx]

**Revision of Manuscript #** **PBIOLOGY-D-21-02477R1 entitled “Hasty sensorimotor decisions rely on an overlap of broad and selective changes in motor activity” by Gerard Derosiere, David Thura, Paul Cisek and Julie Duque.**

We addressed reviewers’ comments carefully and we believe that the recommended changes have allowed us to improve significantly the manuscript. We hope the modifications will satisfy the reviewers and the editors as well. In the revised manuscript, we highlighted in blue the main changes as well as specific passages relevant to our replies below. Please find below our detailed responses to the reviewers’ comments.

**Reviewer #1**

This is an impressive paper. It rarely happens that I have no 'major comments' on a manuscript, but this paper is meticulous in all regards, and the results are very interesting and convincing. It is very well written and suited for this journal as well.

**Authors’ answer**: Thank you very much for the overall enthusiasm on our article.

My only minor comments are:

Line 206: is the word 'in' missing here?

**Authors’ answer**: Yes indeed. This was corrected.

A reference to Figure 6.C seems to be missing

**Authors’ answer**: Right. We now refer to Figure 6.C, lines 489.

Line 822: should 30% be 40%?

**Authors’ answer**: We are referring here to the percentage of easy and misleading trials, which was 30 % in total. We clarified this point in the text (lines 911-912).

Line 896: change "between each block" to "between blocks"?

**Authors’ answer**: Done.

Line 1069-1070: "…, we determined the level of sensory evidence at the time of commitment (i.e., at DT)." This did not make sense to me, as DT is a duration and not a time point. If I understand things correctly, then the time point that the authors refer to here is [time of jump1] + [time of sensory processing] + DT

**Authors’ answer**: Reviewer is right that this information was unclear in the former version of the manuscript. We edited this paragraph accordingly and removed the confusing allusion to DT in brackets (lines 1181-1186).

**Reviewer #2**

Derosiere et al. present data from three samples of participants who made left or right index finger responses based on the slow accumulation of tokens to the left or right of a computer monitor. All participants completed two types of block, varying the punishment for incorrect responses in order to manipulate their internal speed-accuracy tradeoff settings. Two of three groups received concomitant TMS, targeting either the hand or leg motor representation, with MEPs recorded in each case from 3 muscles at four possible times. This provided a somatotopically broad assay of motor activity as pressure (and to a lesser extent evidence) built towards a response. Key findings were a general increase in corticospinal excitability through the trial, with an increased focus on speed being associated with further enhanced excitability of the responding finger and ipsilateral leg, but reduced excitability of ipsilateral task-irrelevant digits. Increased focus on speed was also associated with a reduction in the correlation between MEPs of the responding finger and other digits, although the statistical inference for the latter result is unconventional (and less demanding than is traditional).

This is a well-written paper. In terms of the novelty of the result - I cannot claim anything like exhaustive knowledge of this field, but I know that at least one previous publication (which the authors cite) has investigated how corticospinal excitability (measured via TMS/MEPs) varies across the period of motor preparation alongside a manipulation of the speed-accuracy tradeoff. However, the current work differs from this (and many other) TMS/MEP papers by measuring a large number of task-irrelevant muscles (both close to and far from the task relevant ones) to probe the motor cortex more widely. This is an appealing feature. I personally find the result interesting, and there are implications for neurocomputational theories of action preparation. I imagine there would be some interest both within and outside the immediate field. From a methodological perspective, I have a few queries, but the work seems broadly solid and the conclusions are sensible.

**Authors’ answer**: We would like to thank the reviewer for her/his overall positive opinion on our work.

Essential:

1) The authors' final analysis investigates correlations between MEPs from the responding index finger and other muscles of the body. They then compare these correlations between SAT contexts, finding some significant differences. The problem is that the statistical inference for these results is unconventional, and readers are likely to take them to mean something that they do not. To generate their results, the authors pool MEPs across multiple participants, which violates the assumption of independent data points. This is unlikely to generate spurious correlations, as the MEPs are normalised for each individual before being combined. However, the associated p values (for both the correlations, and the subsequent contrast across contexts) will not mean what most readers will take them to mean. When confronted with a p value from a group analysis, we expect that the p value permits a generalisation from the study sample to the population of all people (or at least all people with similar characteristics to the sample). This is not currently the case for this analysis, and p values will be lower than they would be for the standard generalisation. Instead, the p values reported here reflect a generalisation to all MEPs coming specifically from this particular set of people. In approximate order of satisfactoriness, possible solutions would be: a) running this analysis using the correct multilevel model; b) calculating the correlations for each individual and then comparing the group-average correlations across contexts in a second step; or c) explaining to the reader in the results and/or discussion that p values (and associated significance) are exaggerated relative to a standard group-level statistical analysis.

**Authors’ answer**: Thank you for raising this important issue. We indeed considered performing separate correlations for each subject. However, the number of trials per subject was relatively low (*i.e.*, ~ 19 trials per subject on average), which may have led us to obtain spurious correlations in some subjects. By grouping the trials of all subjects together, our aim was to maximize the number of data points in each correlation (367 trials in total). We acknowledge the generalization issue inherent to this approach and now discuss it in the discussion sections as suggested by the reviewer (lines 667-984). Of note, while we agree that the R- and p-values of each correlation may have been exaggerated using this approach relative to a standard group-level statistical analysis, we applied this procedure for both contexts and the putative exaggeration of statistical values should have thus concerned both contexts. Despite this, we observed significant differences in R-values between contexts, which we quantified using the Fischer’s Z procedure and represents the most important outcome of this whole analysis. This point is also clarified on lines 680-684.

2) The authors perform a fairly elaborate data-matching procedure in order to ensure that RTs were similar for trials included from the two SAT contexts. I understand the concern over matching nearness to the ultimate motor act, but, unless I have misunderstood, I think the procedure could replace one concern with another. Specifically, the adjusted data essentially compares slow trials from the high-pressure condition with fast trials from the low-pressure condition. Despite the fact that evidence is maintained within a fairly ambiguous range across the TMS period (which, by the way, should be emphasised in the results, and not just in the detailed methods) it seems to me that a relative difference in speed of response might reflect systematic differences in the evidence (i.e. number of tokens) accumulated earlier on. Evidence might therefore not be well matched (through randomisation of trials) as it would be in the unselected data. This concern would apply particularly to later TMS times. It matters, because the authors wish to tie their results as far as possible to changes in SAT context (and therefore urgency). Ideally, I would like to see the authors report on the average evidence in favour of the responding digit for different times/contexts based specifically on the selected subsets of trials. Furthermore, if there is any evidence that it differs, I think it would be beneficial to report whether the same results are obtained based on the full (unmatched) data, even if only briefly, e.g. a few sentences linked to a supplementary figure.

**Authors’ answer**: We realize the structure of ambiguous trials was not described with enough details in the former version of the manuscript and was potentially confusing. We thus added further information in the method section related to this aspect (lines 898-904). As highlighted by the reviewer himself, evidence was maintained within a fairly ambiguous range across the TMS period. In fact, in every ambiguous trial, the success probability oscillated between 0.5 (even token dispersion between lateral circles) and 0.66 (*i.e.*, corresponding to a difference of 1 token between lateral circles) until and including Jump_9_. Correspondingly, the evidence oscillated between 0 (even token dispersion between lateral circles) and 0.42 (*i.e.*, 1 token difference between lateral circles) until that jump. After that, from Jump_10_, evidence started to favor one circle over the other (2 or more token difference between the two circles). Importantly, the level of sensory evidence present in the visual display was the same in the two contexts at each TMS timing. This is true for both the RT-matched (as presented in Figure A, below) as well as the full, RT-unmatched dataset of course. Nonetheless, following the reviewer’s comment, we did check whether the effects obtained on motor excitability when using the RT-matched dataset still hold when exploiting the full, RT-unmatched dataset, and reassuringly, this was in fact the case (see Figure B, below, now included in Figure S9). We added a summary of this information in the revised manuscript at lines 394-399.

Figure A: The level of sensory evidence was the same in both contexts in the trials selected for the MEP analysis following the RT-matching procedure.

Figure B: The effects of time and context obtained on motor excitability when using the RT-matched dataset still hold when exploiting the full, RT-unmatched dataset.

3) For the most important experimental manipulation (SAT context) we are told that the order of the sessions was counterbalanced across participants (line 868). However, this cannot be completely true for two out of three groups (which have odd numbers of participants), and, in particular, this may be more substantially incorrect for the MEP analyses, from which a number of participants have been excluded. Failure to fully counterbalance introduces a systematic confound across the main experimental manipulation. Solutions, in approximate order of satisfactoriness, would include: a) increasing the sample to make sure that counterbalancing is complete for all samples from which key results are derived; b) reporting the actual degree of counterbalancing and including block order as a factor in Bayesian ANOVAs to provide evidence favouring the null for associated main effects and interactions; c) reporting the actual degree of counterbalancing and discussing this weakness.

**Authors’ answer**: To ensure that the effects of CONTEXT observed on DT, accuracy, urgency intercept and MEP amplitudes on the chosen side did not depend on the lack of counterbalancing in the corresponding samples, we performed Bayesian ANOVAs, testing whether the factor CONTEXT interacted with SESSION-ORDER. These analyses showed that none of the effects of CONTEXT reported in the manuscript depended on the SESSION-ORDER (see lines 244-253 and 372-380 and supplementary Table S2 and S7). We think this analysis further reinforces the results of the manuscript and thank the reviewer for the suggestion. We now also report the exact number of counterbalancing for each of the main analyses performed in the manuscript (see lines 244-247, 372-376 and 957-958).

4) It may be an oversight on my part, but I saw no explicit mention of the randomisation of the order of trials for non-blocked factors such as TMS timing. This was almost certainly applied, but should nonetheless be clearly stated (as if this was not applied it would be a problematic systematic confound).

**Authors’ answer**: Sorry for this omission. We added this information lines 1093-1094.

5) Key results are analysed using three-factor ANOVAs. I don't have any serious doubts about these results (although I personally would have started from a four-factor ANOVA including response side, in order to make a clearer statistical inference regarding the different patterns obtained for responding and non-responding sides). However, the pipeline for following up interactions from a high-dimensional ANOVA seems non-standard (pg. 13). The authors go straight from a three-way interaction to pairwise t-tests (with Tukey correction, but no indication of how many contrasts are being considered in this correction procedure). A more standard way to follow up the context*timing*representation interaction, based on the way the authors choose to organise the data in Figure 4, would be to next perform three separate two-way (context*timing) ANOVAs, one per digit. It looks likely that a two-way interaction would then be found for the responding index digit, to be followed up by three pairwise tests to reveal the important difference between contexts at token 7 (so no real change there). However, it's less clear that such a 2x3 interaction would emerge for the pinky or thumb. Without it, the pairwise follow ups would be unnecessary and the final result would (most likely) be a main effect of context, i.e. an effect on average across all three times, for these digits. The authors may not like my suggestion to follow a more standard follow-up pipeline, but they should at least state exactly how many (and which) pairwise tests were run and corrected for. They should also make a clear statement that all other main effects and interactions from their three-way ANOVAs were non-significant (or provide a bit more information about this if that is not the case, e.g. the way in which the presentation has been made selective for the purpose of narrative clarity).

**Authors’ answer**: Following the reviewer’s comment, we now provide the exact number of between-pair post-hoc comparisons performed for each significant interaction (*e.g.*, see lines 309 and 347-348). We also mention that lower-level interactions were in some cases significant and report them in the revised manuscript (lines 342-345). However, as sensed by the reviewer, we do not report the post-hoc tests in detail for these lower-level interactions for the purpose of narrative clarity, as mentioned lines 344-345. Finally, in addition to using Tukey’s HSD post-hoc tests, we performed the alternative post-hoc decomposition suggested by the reviewer and report the outcome as a supplementary table (*i.e.*, Table S7, referred to in the text at line 365).

Additional:

Page 9 - the authors assess the behavioural SAT in the entire sample, which is reasonable. However, they go on to view effects on corticospinal excitability in a selected sample that is not likely to be random (relative to the overall sample) in terms of its experience of a SAT. Specifically, it seems likely that the participants who get excluded (as a result of RT matching) would mainly be those who have the largest behavioural SAT. I would therefore suggest that the behavioural results demonstrating a SAT be run in just the included samples from later analyses, as a supplementary figure or similar.

**Authors’ answer**: Thank you for this suggestion. We performed this additional analysis and added a supplementary figure (Figure S5), showing that the subjects included in the MEP analysis exhibited a significant effect of context on DT, accuracy and urgency. We refer to this additional analysis in the revised manuscript, lines 297-299.

"251 Further, a Bayes Factor analysis provided evidence for a lack of effect of the subgroup on all of these behavioral data." I appreciate that full stats are provided in the supplementary materials, but it feels odd not to have anything at all here (e.g. the range of Bayes factors observed). More generally, for the Bayesian statistics, the authors should say something about the assumptions made regarding the alternative hypothesis (e.g. what was the prior on effect size) or at least identify the software/routine they used, stating that default settings were used if that is the case.

**Authors’ answer**: We now provide details regarding the Bayes factors obtained for the lack of effect of TMS-SUBGROUP, line 261. Further, we added details regarding the toolbox and routine exploited on lines 1431-1433.

"288 Normalized MEP amplitudes displayed a main effect of TIMING in TMSFinger subjects (i.e., Jump1 vs Jump4 vs Jump7 in a repeated-measures [rm]ANOVA)." It would be helpful to the reader (mainly for anticipating what is to come) to start out by stating that this result comes from a three-way ANOVA with factors timing, context and representation (although I appreciate that this info appears in the methods).

**Authors’ answer**: Done, lines 303-306.

Discussion section. The authors don't seem to have pointed out any weaknesses or limitations to their work. While understandable (if not condonable) from a strategic perspective, this is poor scientific practice.

**Authors’ answer**: The reviewer is certainly correct that every study has limitations that should be explicitly clarified. We now dedicate a paragraph to some limitations of our study in the discussion section (please see lines 667-684).

Lines 545-556. Is it also worth considering functional/anatomical coupling between muscles? This would seem a particular feature of the motor system compared to sensory systems (i.e. to generate one action we almost never utilise a single muscle in isolation, although that would be more plausible here than in more natural contexts requiring stabilising postural responses and so forth).

**Authors’ answer**: This is a good suggestion. We’ve added the following text (lines 606-608): “Such a mechanism may be of particular importance in the motor system, where populations of corticospinal cells projecting to different muscles may be recruited concurrently during action execution.”

Methods section - it would be helpful to say something about the basis for selecting the size of the three samples. It would also be helpful to say something about the degree to which analyses were developed a priori vs. post hoc. For example, in my experience, in the absence of pre-registration it is typical to have a broad sense of the intended analyses (e.g. mean differences in MEPs will be assessed via ANOVA) without precisely mapping out all the steps (e.g. the thresholds for exclusion of trials/subjects; the factors included in the ANOVAs vs. treated in separate ANOVAs).

**Authors’ answer**: Thank you for this suggestion. We have added more details on this aspect in the revised manuscript, lines 835-838, 1420-1422 and 1432-1433.

The information about the number of trials of each kind (e.g. pg. 36) is rather difficult to digest. Could the authors present a table to help with this, e.g. showing the number of trials per block for each intersection of design-level factors?

**Authors’ answer**: We added a table a recommended, lines 1118-1132 of the revised manuscript.

1157 "In order to prevent contamination of the measurements from background muscular activity, trials in which the root mean square of the EMG signal exceeded 3 SD above the mean before stimulation (i.e., -250 to -50 ms from the pulse) were discarded from the analyses (rejection rate: 8.48 ± 0.43 % in TMSFinger subjects and 0.99 ± 0.05 % in TMSLeg subjects)." Would this procedure not leave participants with preactivation on the majority of trials relatively untouched? Were there any such participants?

**Authors’ answer**: None of the participants presented such a continuous preactivation as they were asked to relax if any contraction was detected by the experimenters, who screened the EMG signal visually throughout the acquisitions. We thus believe it is quite unlikely that the procedure exploited here left some participants with background activity, even if possible in theory. Information related to this concern is now provided lines 981-985, 1145-1146 and 1274-1277.

1255 "To this aim, we exploited the single-trial MEPs obtained at Jump7 following the 100 iterations of the RT-matching procedure described above. We first normalized single trial MEPs as a percentage of the average baseline amplitude for each finger representation, each timing and each context and then computed Pearson's correlations between the five pairs of muscles for each timing and each context." Is there a typo here? If not, how can this have been done for each timing when only jump7 trials are included?

**Authors’ answer**: Yes, there was a typo here. We edited the text accordingly (lines 1376-1381). Thank you for pointing it out.

**Reviewer #3**

This study tests the hypothesis that speed-accuracy tradeoff (SAT) relies on influences exerting broad changes on the motor system - i.e., increasing the activity of the motor system globally in hasty actions. Fifty subjects were trained to perform a perceptual decision task, in which 15 tokens jump one-by-one every 200 ms from a central circle to one of two lateral target circles. The subjects had to choose which of the targets will receive the majority of tokes by pressing a keyboard key using left (for the left target) or right (for the right target) index finger. Subjects were free to respond at any time from Jump-1 to Jump-15. Correct trials led to reward, whereas incorrect trials to penalties. The reward decreased over the course of the trial, producing an increasing urge to decide. In one block of trials, incorrect decisions were severely penalized (-14 cents), emphasizing the need for cautiousness. However, on another block of trials, the penalty of incorrect decisions was only -4 cents, encouraging subjects to make hasty decisions. Transcranial Magnetic Stimulation (TMS) was applied on multiple motor representations of the motor cortex to elicit motor evoked potentials (MEP) in fine finger and leg muscles. The results showed that hasty decisions cause a broad amplification of motor activity but only to the chosen side. Importantly, the motor activity of the areas surrounding the choses index representation was suppressed in hasty decisions. This is an interesting study that explores a very important topic in motor control, and fits within the scope of PloS Biology. However, there are some issues that the authors need to address before the manuscript is considered for publication.

**Authors’ answer**: We would like to thank the reviewer for her/his positive comments on our study.

Major issues

1. The study uses TMS to elicit motor evoked potential (MEP) in nine finger and leg muscles. There are many studies that report strong variability of the MEP responses induced by TMS [1]. Although this variability can be generated by technical factors, such as the location, orientation and stability of the coils, this variability remains even after controlling these factors. Some studies showed that this inherent variability can result from neurophysiological changes in the CSE pathway. Some studies remove the first few MEPs, since most of the variability is expected in the first few MEPs due the regional changes of the blood flow [3], although current study argues that even removing the first few MEPs does not further increase the reliability of MEP responses. So, how did the author deal with this inherent pitfall of the TMS to induce MEP responses?

**Authors’ answer**: This is an important point. We provide information related to this concern in the revised manuscript lines 1108-1132 and lines 1281-1283. First, to attenuate any putative effect of MEP variability on our measures, outlying MEP values – with an amplitude exceeding 3 SD around the mean of the corresponding condition – were excluded from our analysis (see lines 1281-1283). Second, a quite high number of MEPs was acquired for each context (as now clarified lines 1112-1129), with 170 MEPs elicited in ambiguous trials per context, among which 58 were obtained at Jump_1_, 56 at Jump_4_ and 56 at Jump_7_. These high numbers are quite unusual for TMS studies (*i.e.*, which typically involve 10 to 25 MEPs per condition). Having a large number of MEPs reduces within-subject variability when computing the SD in the analysis described above, making this analysis more likely to exclude outlying MEP values. Finally, an analysis performed on a former MEP dataset showed that the coefficient of variation of MEP amplitudes (*i.e.*, the SD normalized over the mean amplitude) does not vary according to SAT context, suggesting that MEP variability was likely to be comparable in the two contexts here.

2. One of the key findings of this study is that the hasty context was associated with particularly large MEPs, including in the leg muscles, though this effect was limited to the chosen side. So, if hasty actions affect both fingers and leg muscles, it could be argued that the effects are related to the attentional mechanisms - i.e., hasty actions require increase of attention for selecting the correct options. In other words, since the effects are non-effector specific, then we cannot exclude the hypothesis that large MEPs are associated with increase on the participants' attention. The authors need to discuss this alternative hypothesis.

**Authors’ answer**: We understand the reviewer’s point. Yet, we believe it is unlikely given the lateralized nature of this MEP increase and the fact that it only emerged at jump_7_ in the index finger representation. Note also that the validity of applying the concept of “attention” to contexts such as this is quite debated at the moment (*e.g.*, Poldrack 2011; Hommel et al. 2019) and we feel it would be confusing to discuss our data following this line. We can reconsider our point if the reviewer feels strongly about it.

3. How can the affordance competition hypothesis, which was proposed by Paul Cisek (one of the authors of the manuscript) explain the effects of the context decision (hasty vs. caution) to the MEPs?

**Authors’ answer**: We frame our study by referring to sensorimotor theories of decision-making and to the idea of a competition between motor representations at lines 65-70 of the revised manuscript. As such, SAT context can be seen as a factor that biases the competition occurring within motor representations. Specifically, our findings show that hastiness is associated with an amplification of motor activity in the chosen hemisphere as the choice unfolds, ensuring a faster rise-to-threshold of neural activity in this hemisphere. This idea is now discussed lines 571-574.

4. Is it possible the TMS to cause any changes on the decision-making process? Previous studies have showed that "temporal lesions" from TMS can bias the decision or change reaction time and other characteristics of the decision-making process. How does the author ensure that TMS did affect the decision process?

**Authors’ answer**: This is a potential issue to consider indeed, thank you for pointing it out. The revised manuscript contains information related to this concern at lines 189-191, lines 254-264, as well as in supplementary Figure 2. In fact, we exploited TMS over the finger and the leg representations in two subgroups involving 21 and 22 subjects (*i.e.*, TMS_Finger_ and TMS_Leg_ subjects, respectively), and also included a subgroup of No-TMS subjects (n = 7; *i.e.*, without stimulation), allowing us to control for any effect of TMS on decision behavior.

Importantly, we analyzed the DTs, accuracy, as well as the slope and intercept of the urgency functions while considering the TMS subgroup (*i.e.*, TMS_Finger_, TMS_Leg_, No-TMS subjects) as a categorical predictor in our analyses (*i.e.*, using ANOVAs). As evident in the Figure C below (Figure S2), we did not find any significant effect of the TMS subgroup on the behavioral data, nor of its interaction with the factor CONTEXT (*i.e.*, hasty vs cautious context). Further, a Bayes Factor analysis provided strong evidence for a lack of effect of the subgroup on all of these behavioral data (all Bayes factors ranged between 4.06 and 8.35). In fact, the three subgroups presented very similar effects of context on decision behavior. This indicates that the application of TMS over the finger or leg representations did not perturb the decision process in this task. This finding seems consistent with the results of a former study from our group, showing that a disruption of M1 activity using a repetitive TMS protocol does not perturb decision speed, accuracy and urgency in this task (Derosiere et al., 2019).

Figure C: Decision behavior was comparable in the three TMS subgroups (*i.e.*, TMS_Finger_ [top panel], TMS_Leg_ [middle panel] and No-TMS subjects [bottom panel]).

5. How did they ensure that they targeted the same motor areas with the same direction across all subjects?

**Authors’ answer**: Information related to the coil positioning, orientation and current direction is now provided lines 1002-1005, 1028-1030, 1039-1041, 1051-1055, ans 1065-1097.

We adopted the same procedure for every subject. In TMS_Finger_ subjects, the two figure-of-eight coils were placed tangentially on the left and right side of the scalp with the handles oriented towards the back of the head and laterally at a 45° angle away from the midline (see Figure 1.C). We exploited a monophasic pulse eliciting a current with a postero-anterior direction in the cortex. In TMS_Leg_ subjects, the batwing coil was initially placed tangentially on the vertex of the scalp with the handle oriented towards the back of the head and parallel to the midline. Then, we turned the handle incrementally following an anticlockwise direction in order to orient the magnetic field towards the leg representation in the left M1 and to obtain maximal MEP amplitudes in the right leg muscles. We exploited a biphasic pulse, which was set such that its first half also elicited a current with a postero-anterior direction in the cortex. In both subgroups of subjects, the coil sites were marked on an electroencephalography cap fitted on the subject’s head to provide a reference point throughout the experimental session.

Minor issue

1. How did they select the four different time instance (baseline, 1, 4, 7) to perform the TMS? What was the criterion?

**Authors’ answer**: We now provide information on this point lines 1112-1013. As such, ambiguous trials are characterized by a distribution of tokens that remains balanced across the two lateral circles until Jump_10_. Thus, most responses occurred after Jump_7_ in such trials, allowing us to obtain a large proportion of MEPs before the subject made her/his decision when stimulating at Jump_1_, Jump_4_ and Jump_7_.

2. Was it any difference on the MEP responses between correct and incorrect trials?

**Authors’ answer**: As such, subjects made on average 15 % of incorrect choices. Hence, a too small sample of MEPs was obtained in incorrect trials per timing and context, preventing us to address this question in the present study. We now highlight this aspect on lines 1269-1271.

**References**

Derosiere, G., Thura, D., Cisek, P., & Duque, J. (2019). Motor cortex disruption delays motor processes but not deliberation about action choices. Journal of neurophysiology, 122(4), 1566-1577.

Hommel, B., Chapman, C. S., Cisek, P., Neyedli, H. F., Song, J. H., & Welsh, T. N. (2019). No one knows what attention is. Attention, Perception, & Psychophysics 81 (7), 2288-2303.

Poldrack, R. A. (2011). Inferring mental states from neuroimaging data: from reverse inference to large-scale decoding. Neuron, 72(5), 692-697.
