# Supplementary material for: Hasty sensorimotor decisions rely on an overlap of broad and selective changes in motor activity
Source: PLoS Biol. 2022 Apr 7;20(4):e3001598. doi: 10.1371/journal.pbio.3001598 (PMC9017893; doi:10.1371/journal.pbio.3001598)
Supplement: Attachment — Submitted filename: Derosiere_Thura_Cisek_Duque_Rebuttal_MinorRevision.docx [file pbio.3001598.s013.docx]

**Minor revision of Manuscript #PBIOLOGY-D-21-02477R2 entitled “Hasty sensorimotor decisions rely on an overlap of broad and selective changes in motor activity” by Gerard Derosiere, David Thura, Paul Cisek and Julie Duque.**

**Editor’s comments**

Based on the reviews, we will probably accept this manuscript for publication, provided you satisfactorily address the remaining points raised by reviewer 2. The Academic Editor thinks that the statistical concern is justified and that it would be the preferable solution if you could fix it with a multilevel statistical model to distinguish within from between subject variance, or else work around it with one of the strategies suggested by the reviewer.

**Authors’ answer**: Thank you for considering this article for publication in PLOS Biology. We have followed the suggestion of Reviewer 2 and of the Academic Editor by replacing the former simple correlation analysis by a repeated-measures correlation. Repeated-measures correlation does not violate the assumption of independence of observations and distinguishes within- from between- subject variance. Importantly, while this new correlation analysis yielded slightly different R- and p-values relative to the former analysis, the results still support the conclusion of a decorrelation of activity changes from the cautious to the hasty decision context. All of the other minor editorial requests have been addressed, as detailed in the cover letter.

**Reviewer #2**

The authors have responded appropriately to the majority of my concerns and the paper is nearly ready for publication. However, I need them to make a further adjustment in relation to essential point #1 from my original review.

The authors have opted to address this point by adding a caveat paragraph in the discussion. Most of this is fine, but they conclude it with (line 680):

"However, this procedure was applied for both contexts and the putative exaggeration of statistical values, if any, should have thus concerned both contexts. Despite this, we observed differences in R-values between contexts, which represent the most important findings of this whole correlation analysis."

Unfortunately, the issue I described relates to the p values for both the correlations themselves, and the test of differences between correlations (i.e. Fisher's z procedure). Fisher's z procedure produces a statistic based on r that is normally distributed (and thus suitable for a subsequent t-test) but the standard error of this statistic (i.e. the essential component that normalises group differences to determine p values) is determined using the sample size (N) under the assumption of independent data points. The authors are, I assume, providing an N of 367 for this calculation (or, more probably, giving their software 367 rows of data so that it will assume a sample size of 367). But of course, they only really have 19 participants. A standard error based on a sample size of 367 will be about four times smaller than one based on a sample size of 19, yielding, for example, a t statistic that is about four times larger. This is what I mean when I say they underestimate their p values (in relation to a typical generalisation to a population of all people). As I mentioned in my original review, the best solution would be to use a multilevel model or two-step procedure that will yield p values with the standard meaning. If this is not done, the authors need to be unequivocal in describing this limitation (or else explain how/why I have misunderstood their Fisher's z procedure in their reply).

**Authors’ answer**: We have followed the reviewer’s suggestion and have replaced the former simple correlation analysis by a repeated-measures correlation (rmCorr; Bakdash, J.Z., and Marusich, L.R. (2017). Repeated measures correlation. Front. Psychol. 8, 456), as described at lines 472-508 and 1391-1409. RmCorr accounts for non-independence among observations using analysis of covariance (ANCOVA) to adjust statistically for inter-individual variability. By removing measured variance between-participants, rmCorr provides the best linear fit for each participant using parallel regression lines (the same slope) with varying intercepts, as presented in Figure S10. Hence, this approach does not violate the assumption of independence of observations and distinguishes within- from between- subject variance. Further, we have decided to replace the Fisher’s Z procedure by a direct comparison of the 95 % confidence intervals (CIs) calculated using the rmCorr approach. A difference in R-values between contexts was considered as significant when the 95 % CIs for the compared R-values did not overlap.

As predicted by the reviewer, some of the R-values were slightly lower when using this new approach than when using the former simple correlation analysis. However, the results still support the conclusion of a decorrelation of activity changes from the cautious to the hasty decision context.

Note that while all correlations were positive using the former analysis, two of them were negative using the rmCorr analysis. These negative correlations were present in the hasty context, in accordance with our interpretation of a decrease in positive correlation in this context. Given the presence of negative correlations, we have removed the former panel B. of Figure 6, which presented the p-values of the correlations as a way to compare the significance of former positive correlations. We think that presenting together p-values for positive and negative correlations in a single graph would now be misleading. Instead, we now provide relevant p-values for positive correlations in the main text.

Altogether, we think that these modifications answer the main concern raised above. We thank you again for all your suggestions, which we think have allowed us to improve substantially the manuscript.
